# Factors predicting mental health in youth during the first COVID-19 lockdown in Spain

**DOI:** 10.1186/s40359-023-01367-0

**Published:** 2023-10-06

**Authors:** Isabel Vicario-Molina, Eva González Ortega, Ruth Pinedo González, Andrés Palacios Picos

**Affiliations:** 1 Department of Developmental and Educational Psychology, University of Salamanca, Avenida de la Merced s/n, Salamanca, 37005 Spain; 2 Department of Psychology, University of Valladolid, Plaza de la Universidad, 1, Segovia, 400005 Spain

**Keywords:** COVID-19, Lockdown, Mental health, Risk factors, Protective factors

## Abstract

**Background:**

The first COVID-19 lockdown decreed in Spain by the government resulted in a significant disruption in the daily lives of youth that may have affected their mental health. The aim of this study was to identify factors that influenced youth mental health during this period. Methods: Between March and May 2020, a sample of 1205 youths (age range 18–24 years) from across Spain completed a questionnaire that assessed sociodemographic, mental health, loneliness, coping with humour and healthy habits. Data were analysed using Structural Equation Modelling.

**Results:**

The proposed model showed good fit values, and significant variables explained 37% of mental health. loneliness, coping humour, healthy and unhealthy habits, and gender significantly predicted youth mental health.

**Conclusions:**

The consequences of the pandemic and confinement derived from COVID-19 seemed to be especially severe for youth, increasing their mental health vulnerability. It is recommended that evaluating the long-term effects of confinement on this population, the mental health needs they may have and the support resources that would help meet them in situations of isolation, paying special attention to females.

**Supplementary Information:**

The online version contains supplementary material available at 10.1186/s40359-023-01367-0.

## Introduction

On January 30, 2020, the World Health Organization declared the COVID-19 a public health emergency of international concern that lasted until May 5, 2023. The coronavirus pandemic has posed a huge challenge to the world, with nearly 7 million deaths [1]. On March 14, 2020, Spain declared a state of emergency, which lasted until June 21 [2]. This lockdown, the strictest in Europe [3, 4], was applied uniformly throughout Spain. This measure helped to control the pandemic, but resulted in a significant disruption in the daily lives of many youths. One of the main characteristics of this developmental stage is the growing importance of social bonds with peers. Thus, the social isolation caused by the lockdown could have had negative impacts on their development.

### Mental health during the COVID-19 lockdowns

The lockdown measures in Spain may have led to harmful feelings such as loneliness [5, 6] and had significant outcomes on mental health [7, 8] (e.g., anxiety or depression), especially in at-risk groups such as youths [9–11]. While studies are scarce, the available data indicate an increase in youth’s mental healthcare needs during the lockdown period [12]. López-Castro et al., [13] studied NYC university students during lockdowns and found almost 90% exceeding scores for depression and more than 60% experiencing anxiety. Mohler-Kuo et al., [14] also observed that the disruptions in social life and important activities, and the uncertainties related to the lockdown and the pandemic, were common stressors for youth. One-fifth of the sample met the criteria for attention deficit hyperactivity disorder, depression, or generalized anxiety disorder.

Though the impact of the COVID-19 pandemic varied across youth and was extremely challenging for some [15, 16], as in the general population [7], the relationship between confinement and mental health difficulties is likely influenced by several factors, including lifestyle or the ability to cope with isolation [17, 18]. With regard to gender and age, studies have shown being female is a risk factor for developing mental health problems in youths in the context of COVID-19 lockdown [18]: higher levels of stress, anxiety and depressive symptoms [13–15], less self-esteem, more problems with emotional regulation and more somatic complaints [19] have also been identified.

### Loneliness during COVID-19 lockdowns

At a stage of development where social contact and interactions are so crucial, the loneliness associated with confinement could have a significant negative impact on the mental health of youth. In fact, this age group has been identified as being particularly vulnerable to feelings of loneliness [20]. Perlman and Peplau [21] define loneliness as “the unpleasant experience that occurs when a person’s network of social relations is deficient in some important way, either quantitative or qualitative” (p. 31).

The impact of loneliness on mental health in youths has received significant empirical support [22, 23]. Studies conducted during the COVID-19 pandemic have found a relationship between loneliness and mental symptomatology, especially depression and sadness [24–27]; however, the relationship with anxiety is not clear [24, 26]. It seems that the duration of the feelings of loneliness is of more consequence than the intensity [26]. In addition, the relationship between loneliness and mental health is influenced by age, where youth are more vulnerable than middle-aged or older people [12, 17].

### Coping and healthy habits during the COVID-19 crisis

According to the literature, coping strategies helped to prevent loneliness and depression [28], and hindered increases in substance use and mental health problems [29] among youth during the first months of the pandemic. Likewise, the subjective well-being of youths was fostered by the implementation of positive [30] and adaptive coping strategies, such as physical activity, daily routines, structured activities, developing new interests, and a positive reading of the ongoing period [31].

Regarding healthy habits, previous literature suggests that the lockdown period was characterized by an increase in the exposure to screen-based devices [32–34], food intake [32, 34], and by a decrease in physical activity and more time of lazing around [32, 34], among youths. An increase in substance use (i.e. cigarettes, alcohol, cannabis) has also been observed [25, 35] especially among females [35].

Social isolation caused by COVID19-derived confinement could have a negative impact on the youth’s mental health and lead to harmful feelings such as loneliness [5, 6]. Therefore, it is particularly relevant to know the impact of confinement decreed in Spain on the mental health of youth, and what factors contribute to explain it. This can help us design specialized interventions targeted at high-risk groups. Furthermore, nowadays this study continues to be useful from a health, social and scientific point of view because the impact of confinement is likely to be prolonged over time and may still affect youth’s mental health [36].

### The current study

This study tested a model to explain youth mental health during the first COVID-19 lockdown in Spain. It was hypothesized that:

- Psychological variables like feelings of loneliness and coping with humour predict youth mental health, inversely and directly.
- Personal variables like gender or healthy habits predict youth mental health. Being female and unhealthy habits predict poorer mental health.
- Contextual variables like area of residence, days of confinement or number of people living in the household are less predictive of youth mental health.

## Materials and methods

### Participants and procedure

The sample consisted of 1205 Spaniards aged between 18 and 24 years. The majority of the participants were female (n = 904; 75%) and had higher education (n = 1060; 88%), and most were students during the lockdown (n = 1036; 86%). Almost 70% lived in an urban area and the majority resided in the regions of Castilla y León (78.5%) and Madrid (6.3%) (see Supplementary material).

After obtaining ethical and data protection approval from the Research Ethics Committee at the University of Valladolid and the Ethics Committee of the Regional Health System (SACYL), an online survey was designed using Google Forms. Non-probability virtual snowball sampling was conducted by sending the link to the survey via email and social networks. Informed consent was obtained through a mandatory question at the beginning of the survey. Data were collected from across Spain during the first lockdown that took place in the months of March (n = 1268; 35.1%), April (n = 747; 21.3%) and May (n = 1493; 42.6%) 2020.

### Measures

Data were collected through an online survey designed ad hoc, which included questions and scales on the following variables:

- Sociodemographic data: Questions with regard to gender, age, educational level, employment status, people living in the household (number and type of relationship), and the area of residence (rural vs. urban) (See Table 1).
- Healthy and unhealthy habits: Five questions about the degree of practice of healthy (healthy diet, exercise) and unhealthy behaviors (smoking, drinking alcohol, taking sleeping pills) during lockdown (from 1 = *none* to 4 = *high*).
- Loneliness: The Spanish version [37] of the Social and Emotional Loneliness Scale for Adults-Short version (SELSA-S) [22] assessed the experience of loneliness, considering social, family, and romantic/couple dimensions (e.g., I feel part of a group of friends; I feel close to my family; I have a romantic or marital partner who gives me the support and encouragement I need). These three subscales have shown a high internal consistency and construct validity [37]. Responses were scored on a 4-point Likert-type scale from 1 (*strongly disagree*) to 4 (*strongly agree*).
- Coping humour: The Spanish version [38] of the Coping Humour Scale (CHS-5) [39] evaluated the extent to which participants used humour in coping with stress in their lives. The scale has adequate reliability and validity according to the authors and is composed of five items (see Table 2). Responses were scored from 1 (*strongly disagree*) to 4 (*strongly agree*).
- Mental Health: The Spanish version [40] of the Mental Health Inventory-5 (MHI-5) [41] assessed psychological well-being and distress through five items (presented in Table 2) with good internal consistency. Responses ranged from 1 (*never*) to 5 (*almost always*).

**Table 1:** Descriptive statistics of sociodemographics for participants during the first COVID-19 lockdown in Spain

Sociodemographics		N	%
*Gender*	Male	301	25
	Female	904	75
*Educational level*	Less than high school	1	0.08
	High school/ Bachelors	144	11.95
	Graduate Degree	1060	87.97
*Who do you live with currently?*	Partner	54	4.50
	Partner and children	1	0.10
	Father/Mother/Siblings	1009	83.70
	Other	141	11.70
*How many people, including you, are currently living in your household?*	1 person	22	1.83
	2 persons	139	11.58
	3 persons	352	29.33
	4 persons	521	43.42
	5 persons	139	11.58
	6 persons o +	27	2.25
*Area of residence*	Urban	839	69.70
	Rural	365	30.30
*Have you set yourself a schedule to plan and organize each day?*	Yes	714	59.30
	No	491	40.70
*How many days have you been in confinement?*	Up to 7 days	117	9.77
	8–24	149	12.44
	25–44	209	17.45
	45–58	357	29.80
	59 or more	366	30.55
*Disability*	Yes	17	1.40
	No	1179	98.60
*Occupation during confinement*	Employed	145	12
	Unemployed	24	2
	Student	1036	86

**Table 2:** Evaluation of the assessment instrument: reliability of the indicators and constructs

	*Item*	*Outer Loading*	*Composite Reliability*	*AVE*
*Mental health*	*P26.1_R.- How much time, during the last month……have you been a very nervous person? (recoded)*	0.77	0.90	0.65
	*P26.2.-…have you felt calm and peaceful?*	0.80		
	*P26.3_R.-…have you felt downhearted and blue? (recoded)*	0.85		
	*P26.4.-…have you been a happy person?*	0.78		
	*P26.5_R.-…have you felt so down in the dumps that nothing could cheer you up? (recoded)*	0.83		
*Healthy habits*	*P17.1.- Healthy diet (no excessive fat or sugar)*	0.86	0.80	0.67
	*P17.2.- Physical exercise (daily)*	0.78		
*No healthy habits*	*P17.3 Smoking*	0.44	0.78	0.57
	*P17.4 Drink alcohol*	0.84		
	*P17.5 Taking sleeping pills*	0.89		
*Coping humour*	*P25.1.- I have often found that my problems have been greatly reduced when I have tried to find something funny in them*	0.70	0.86	0.56
	*P25.2.- I have often felt that if I am in a situation where I have to either cry or laugh, it’s better to laugh*	0.64		
	*P25.3.- Many times I have felt that, in situations that are either to laugh or to cry, I prefer to laugh*	0.77		
	*P25.4.- I can usually find something to laugh or joke about even in crying situations*	0.80		
	*P25.5.- It has been my experience that humor is often a very effective way of coping with problems*	0.83		
*Feelings of loneliness*	*P27.1.- Social Loneliness*	0.90	0.87	0.68
	*P27.2.- Family Loneliness*	0.80		
	*P27.3.- Romantic/couple loneliness*	0.77		
*People living in the household*	*How many people, including you, are currently living in your household?*	1^†^	1	1
*Days of confinement*	*How many days have you been in confinement?*	1	1	1
*Area of residence*	*Where do you live during the confinement?* *Rural area (1); Urban area (2)*	1	1	1
*Gender*	*Male (1); Female (2)*	1	1	1

^*†*^ *For variables with only one indicator the value obtained is always 1.*

### Data analysis

First, descriptive statistics of the sociodemographics of the respondents were obtained. Next, the frequency distributions of the variables were calculated, and their most relevant characteristics were analysed (including normality). Non-parametric tests, such as the Kruskal-Wallis H test, were also performed to establish possible differences in means. SPSS v. 26.0 was used to perform these data analyses. Following these analyses, structural equation modelling (SEM) based on the analysis of variance (partial least squares [PLS]) was performed. This measurement model assesses the quality and the specific contribution of each item to the measurement of the construct (i.e., the relationships between the latent variables and the observed variables), which allows us to assess the psychometric characteristics of the measures.

The relevance of the model was determined, testing the degree of fit in predicting the values of the endogenous constructs and the hypotheses [42]. SEM is considered a second-generation multivariate analysis and is especially recommended when the theoretical knowledge of the problem is limited, the objective is to develop a causal-predictive analysis, or the research questions are complex [43]. Smart-PLS v. 3.3. was used to perform this analysis and statistical significance was set at ≤ 0.05.

## Results

### Descriptive analyses

The sociodemographic characteristics of participants are shown in Table 1. Mental health had a positive asymmetric distribution (0.13), with a mean of 17.85 and a standard deviation of 5.40. No significant differences were found by age in the levels of perceived mental health (H = 13.68; p > .05).

To examine possible multicollinearity, the correlation matrix of the quantitative variables in the final model is presented (Table 3). In addition to these variables, the final predictive model includes nominal or ordinal variables whose values were obtained by means of closed-ended questions from the questionnaire (people living in the household, days of confinement, area of residence, gender).

**Table 3:** Correlation matrix of the quantitative variables of the model

	*Mean*	*SD*	*Mental Health*	*Loneliness*	*Coping*	*Healthy habits*	*No healthy habits*
*Mental health*	17.84	5.40	1				
*Loneliness*	20.74	5.58	− 0.495^**^	1			
*Coping humour*	12.19	3.70	0.396^**^	− 0.158^**^	1		
*Healthy behaviors*	5.07	1.48	0.183^**^	− 0.163^**^	0.156^**^	1	
*Unhealthy behaviors*	3.72	1.10	− 0.119^**^	0.064^*^	0.004	− 0.037	1

**. Correlation is significant at the 0.01 level (bilateral); *. Correlation is significant at the 0.05 level (bilateral).

### Measurement model

The psychometric quality of the indicators and their constructs in the structural equations is specified in their measurement models. The *reliability of the indicators* is obtained through the standardized loadings (outer loading) or the simple correlations of these indicators with their constructs. These loadings are considered adequate when they reach a value above 0 [44]. In our case (Table 2), only indicators P17.3 and P25.2 had values below this level, but it was decided to keep them in the model because of their contribution to the rest of the psychometric values, and because there is enough theoretical justification for their inclusion in the measurement of the construct.

The *reliability of the constructs* is calculated from their internal consistency using the composite reliability indices (*composite reliability*). Values above 0.70 and, more restrictively, above 0.80 are considered adequate [45]. *Convergent validity* should be interpreted as the amount of variance of a construct that is explained by its indicators, and it is obtained from the *average variance extracted* (AVE). In this case, values above 0.50 are admissible [46]. In our measurement model, all the constructs reach values greater than 0.50 (Table 2).

One of the most widely used procedures to ensure discriminant validity is that proposed by Fornell and Larcker [46], who maintain that a construct has this validity if the variance of its indicators is greater than the correlation of the construct with the rest of the variables. In this case (Table 4), the AVE values in the scale itself are greater than that calculated for the rest of the constructs.

**Table 4:** Discriminant validity of the constructs included in the model

	*1*	*2*	*3*	*4*	*5*	*6*	*7*	*8*	*9*
*1.- Coping humor*	**0.75** ^†^								
*2.- No healthy habits*	− 0.06	**0.75** ^†^							
*3.- Healthy habits*	0.16	− 0.06	**0.82** ^†^						
*4.- Gender*	− 0.21	0.07	− 0.06	**1.00** ^†^					
*5.- Days of confinement*	− 0.19	0.03	− 0.01	− 0.01	**1.00** ^†^				
*6.- People living in the household*	− 0.08	− 0.05	0.04	0.01	0.03	**1,00** ^†^			
*7.- Area of residence*	− 0.04	− 0.07	− 0.02	0.05	0.04	0.10	**1.00** ^†^		
*8.- Mental health*	0.36	− 0.21	0.20	− 0.19	− 0.21	− 0.05	0.00	**0.81** ^†^	
*9.- Feelings of loneliness*	− 0.20	0.09	− 0.18	− 0.01	0.03	0.02	0.00	− 0.47	**0.83** ^†^

^*†*^ *Average Variance Extracted (AVE)*

### Structural model (inner model)

Currently, there is a tendency to use measures of general adjustment of the structural model as performed in structural equations based on covariance. Among them, the *standarized root mean square residual* (SRMR) stands out. In this case, values lower than 0.08 are considered indicators of a good fit. In our model, the value of this indicator was 0.06, which indicates a good fit (Table 5).

**Table 5:** Model fit indexes

	*Saturated Model*	*Estimated Model*
*SRMR*	0.06	0.06
*d_ULS*	1.14	1.14
*d_G*	0.49	0.49
*Chi-square*	3343.60	3343.60
*NFI*	0.62	0.62

Once the validity of the measures of the model and the general structural model have been confirmed, it is necessary to evaluate the characteristics of the model and its component elements. To this end, key elements used in this evaluation are: (a) the statistical significance of the path coefficients (β) and the effect size (f^2^), (b) the values of the coefficient of determination (R^2^), and (c) determining the predictive relevance of the model (Q^2^) [47].

**Fig. 1:**
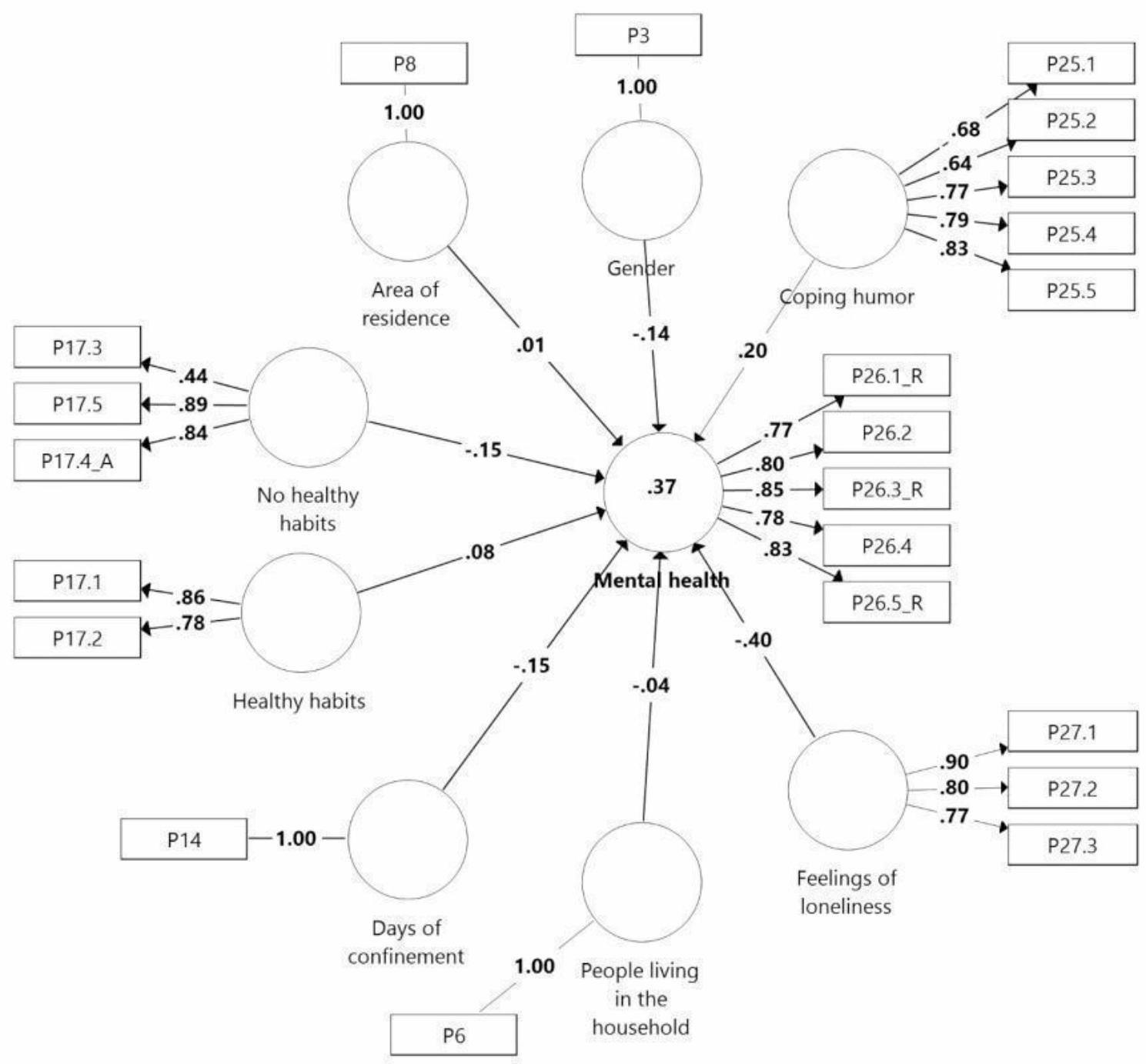
The structural model

The path coefficients of the model allow us to test the initial hypotheses (Fig. 1). Assuming a confidence level of 5%, we can consider all the relationships of the structural model to be significant, except for the variables *area of residence* and *cohabitants.* Of all the significant constructs, the *perception of loneliness* is noteworthy both for the value of its path coefficient and for the size of the effect (β = − 0.40; p = .000; f^2^ = 0.24). In this case, the relationship is inverse; thus, higher levels of loneliness are related to a lower perception of mental health. *Coping strategies* also has a high and significant β coefficient, but a small effect size (β = 0.20; p = .000; f^2^ = 0.06), Therefore, these strategies are a good way to enhance mental health; however, the influence of this variable on mental health is limited.

The two variables related to health habits have different influences on perceived mental health. Specifically, *healthy habits* have a positive influence on mental health (β = − 0.08; p = .002; f^2^ = 0.01). Thus, daily physical exercise increases the perception of health. This is opposed to alcohol consumption or the intake of sleeping pills, which have a negative effect on perceived mental health (β = − 0.15; p = .000; f^2^ = 0.03). The variable *days of confinement* has a significant and inverse relationship with perceived mental health. In this sense, the longer the confinement becomes, the more the perception of health decreases. However, the influence of this variable is limited (β = − 0.15; p = .000; f^2^ = 0.04). Regarding the *gender* variable, the results indicate that young females had a lower perception of mental health than young males during the COVID-19 lockdown (β = − 0.14; p = .000; f^2^ = 0.03).

As noted above, in PLS-SEM models, the predictive quality of the model is determined by the size of its coefficient of determination (R^2^). To consider that a model has predictive ability, the coefficient of determination must be greater than 0.10 [48]. In our case, the value of this coefficient was 0.37, which indicates that the proposed model has a moderate predictive capacity. Thus, despite the complexity of the mental health perception variable, 37% of its variance can be explained by the variables that comprise the final model.

The predictive validity of the model was also confirmed by the Stone-Giesser test (Q^2^). If this test value is greater than 0, then the model has predictive validity [49]. In our case, Q^2^ = 0.23.

## Discussion

As mental health depends on multiple factors and the interactions between them [50, 51], the aim of this study was to investigate whether loneliness, coping humour, healthy and unhealthy habits, gender, days of confinement, the number and type of people living in the household, and the area of residence were associated with mental health in a sample of youths during the first COVID-19 lockdown in Spain.

Considering the factors included in the predictive model, loneliness had the greatest impact on youth’s mental health. Specifically, as indicated by previous literature [16, 20, 24, 52] and hypothesized, higher levels of loneliness predicted a worse perceived mental health status. In youth, the bond and connection with peers is crucial for an integral development [53, 54]. Therefore, based on interpersonal needs theory by Schutz [55], it is logical to expect that a long period of confinement may affect the mental health of this group [6] and be related to health risk behaviors [6]. In fact, the third most significant factor in our predictive model was the number of unhealthy behaviours, such as drug use or taking sleeping pills, which has also shown to have a significant negative impact on the mental health of youths and general population in previous studies [18].

A second significant factor, also as expected, is a humour coping style. It seems that the ability to cope with humour during the pandemic and lockdown predict better mental health [7], as reported in previous studies [56]. This information is useful at a clinical level as it provides a way to empower youth to deal with stressful situations, such as confinement and social isolation.

Finally, the number of days confined or being female were factors that seemed to significantly predict the mental health of youths, although to a lesser extent. Consistently, other research has shown that the mental health of females has been particularly affected by the COVID-19 pandemic [13, 18]. Females appear to be more vulnerable to stressful events and verbalize distress to a greater extent [57], and in contexts of confinement, young females are more likely to regard social isolation as being difficult compared to males [15], and experience higher levels of family responsibilities [58], psychological burden and concern for others [59]. These factors may contribute to explain the gender differences in mental health, but should also be considered elements that generate further social imbalance and inequality between genders.

## Conclusions

The first COVID-19 lockdown period in Spain highlighted the vulnerability of youth people to loneliness, as it had a significant impact on their mental health. According to our data, loneliness had a significant negative impact on their mental health, whereas a positive and humorous coping style and healthy habits were beneficial. Those confined for longer periods and females were also more at risk for mental health problems.

The mental health of youth is thus, at risk in situations of stress and social isolation, such as pandemic confinement. In Spain, youths were not the first age group targeted in the de-escalation measures, and this could have been particularly detrimental to them. We must acknowledge that measures aimed at controlling the virus may have generated new mental health needs that should be expeditiously addressed by the public health system.

In this sense, governments need to be aware and make policies that balance protection against COVID-19 and other possible pandemics with measures to promote youth’s mental health [60]. The lessons learned during the pandemic should be taken into account when designing and prioritizing interventions specific for this population. According to our data, it is urgent to create high-quality plans to detect and treat mental health difficulties. Likewise, it is necessary to develop preventive interventions that reduce risk factors (e.g., loneliness, unhealthy habits) and promote protective factors (e.g., education on effective coping strategies [61] such as humorous coping, as well as on healthy habits). These measures should be accessible and follow a multilevel approach in environments relevant to youth ranging from the media and social networks to the community, education, sports, work and also health systems through primary care workers, thus increasing social support and a sense of connectedness.

In summary, the results of this study have important implications in multiple areas and highlights the need for action to address the mental health of youth in situations of stress and social isolation, including COVID-19, and to promote policies that address the needs of this population group.

Limitations: the sample obtained in the current study may not be representative of Spanish youth as a snowball sampling technique was used to collect data via an online survey.

It is recommended that future studies evaluate the long-term consequences of confinement on the well-being of youth, especially among the female and intersectional identities population, for whom the pandemic may have been particularly challenging. Further research is also needed to address, from the perspective of youths, the needs that they may have and the support resources that would help in situations of confinement or isolation. Such resources are needed to minimise or prevent mental health problems and, in particular, should be tailored towards the most vulnerable.

### Electronic supplementary material

Below is the link to the electronic supplementary material.

Supplementary Material 1

## Data Availability

The datasets used and/or analysed during the current study available from the corresponding author on reasonable request.
