# Supplementary material for: Factors predicting mental health in youth during the first COVID-19 lockdown in Spain
Source: BMC Psychol. 2023 Oct 6;11:317. doi: 10.1186/s40359-023-01367-0 (PMC10559502; doi:10.1186/s40359-023-01367-0)
Supplement: Supplementary file 1 — Supplementary Material 1 [file 40359_2023_1367_MOESM1_ESM.docx]

**Supplementary material**

Table 1. Geographical distribution of the sample

| Autonomous Community in Spain | N | % |
| --- | --- | --- |
| Castilla y León | 868 | 78.5 |
| Madrid | 70 | 6.3 |
| Castilla la Mancha | 20 | 1.8 |
| Galicia | 17 | 1.5 |
| Asturias | 17 | 1.5 |
| País Vasco | 15 | 1.4 |
| Cantabria | 15 | 1.4 |
| Andalucía | 14 | 1.3 |
| C. Valenciana | 12 | 1.1 |
| La Rioja | 12 | 1.1 |
| Aragón | 11 | 1.0 |
| Extremadura | 11 | 1.0 |
| Murcia | 6 | 0.5 |
| Cataluña | 4 | 0.4 |
| Islas Baleares | 4 | 0.4 |
| Islas Canarias | 2 | 0.2 |
| TOTAL | 1106 | 100 |
